# Anal cancer risk and HPV infection knowledge and awareness among Hispanic persons living with HIV in Puerto Rico

**DOI:** 10.21203/rs.3.rs-4565682/v1

**Published:** 2024-06-28

**Authors:** Jessica Hernandez-Marrero, Jeslie M. Ramos-Cartagena, Marievelisse Soto-Salgado, Tanialy Rivera-Santiago, Karen J. Ortiz-Ortiz, Vivian Colón-López, Ashish A. Deshmukh, Ana P. Ortiz

**Affiliations:** University of Puerto Rico Comprehensive Cancer Center, San Juan; University of Puerto Rico Comprehensive Cancer Center, San Juan; University of Puerto Rico Comprehensive Cancer Center, San Juan; University of Puerto Rico/MD Anderson Cancer Center Partnership for Excellence in Cancer Research, San Juan; University of Puerto Rico/MD Anderson Cancer Center Partnership for Excellence in Cancer Research, San Juan; University of Puerto Rico Comprehensive Cancer Center, San Juan; Department of Public Health Sciences, Medical University of South Carolina; University of Puerto Rico Comprehensive Cancer Center, San Juan

**Keywords:** Anal cancer risk, HPV-related cancers, cancer risk knowledge, cancer risk awareness, people living with HIV, Hispanics, Anal cancer prevention

## Abstract

**Background:**

Persons living with HIV (PLWH) have a higher risk of persistent infection with human papillomavirus (HPV) and anal cancer. We evaluated knowledge and awareness of HPV infection and risk factors for anal cancer among PLWH in Puerto Rico (PR).

**Methods:**

Data from a cross-sectional study (2020–2021) were analyzed (n=212). Inclusion criteria included PLWH, aged ≥ 26 years, and living in PR. Telephone interviews collected information on sociodemographic, lifestyle and clinical characteristics. Two 13-item scales were used to assess knowledge of HPV and anal cancer risk factors; adequate knowledge for both scales were defined as scoring >70%. Logistic regression models using generalized linear models were used to determine the association between 1) HPV infection awareness, 2) HPV infection knowledge, and 3) Anal cancer risk factors knowledge.

**Results:**

The median age was 54 years (IQR: 46,58), 67.5% were male, 71.7% reported having an income <$20,000, and 54.3% had an education level of more than high school. HPV awareness was high (82.1%), but only 40.2% and 3.8% had adequate knowledge of HPV and anal cancer risk factors, respectively. In adjusted logistic regression models, men who have sex with men (OR: 1.26, 95%CI: 1.07–1.47) and women (OR: 1.35, 95%CI: 1.15–1.59) aged ≥50 years had higher odds of HPV awareness than heterosexual men in that age group. Moreover, those with history of anal Pap test aged <50 years had more HPV awareness (OR 1.34, 95%CI: 1.08–1.66) than their counterparts. Adequate HPV knowledge was higher among participants with an education level of more than high-school (OR:1.28, 95%CI: 1.10–1.50) and with a history of HPV diagnosis (OR:1.33, 95%CI: 1.08–1.65) than their counterparts. In addition, people with good/very good/excellent health perception had higher odds of HPV knowledge (OR:1.23, 95%CI: 1.03–1.47) than those who reported poor/regular health perception. For anal cancer risk factors, PLWH for ≥15 years had increased odds of having adequate knowledge (OR:1.07, 95%CI: 1.02–1.14) than their counterparts.

**Conclusions:**

Despite high awareness of HPV, limited knowledge about HPV and anal cancer risk factors was observed among PLWH. Results from our study highlight the need for educational efforts within this population as an anal cancer prevention strategy.

## BACKGROUND

People living with human immunodeficiency virus (PLWH) have a higher risk of persistent infection with human papillomavirus (HPV) and consequently anal cancer (AC). HPV infection is the principal risk-factor for AC.(1) The occurrence of this uncommon cancer is gradually increasing. In Puerto Rico (PR), the incidence rate of AC was higher among PLWH than in the general population (27.7 versus 1.1 per 100,000 person-years) from 2000 to 2016.(2) This is also higher compared to the United States (US) national incidence, 1.9 per 100,000 person-year.(3)

AC incidence is associated with HPV risk-factors, such as sexual behaviors, including anal intercourse and multiple sexual partners. Additionally, PLWH, men who have sex with men (MSM), women with HPV-related cervical cancer or precancerous lesions, and immunosuppressed individuals (due to organ transplants or autoimmune disorders) are at high-risk.(4) Furthermore, older age, female sex and smoking are other factors associated with AC.(4)

High-risk individuals must have early diagnosis and screening for AC.(5)

At early stages, AC could be asymptomatic or present with symptoms like other non-serious conditions. These may include anal bleeding, anal or pelvic pain, weight loss, sensation of a mass in the anus or rectum, anal irritation, tissue prolapse, flatus or stool incontinence, and obstipation.(6) The anal Papanicolaou (Pap) test is the principal screening approach recommended for PLWH. This clinical practice was not supported by clinical guidelines(7) until February 2024, when the consensus guidelines of the International Anal Neoplasia Society (IANS) for the screening of AC were published.(5) Integrating this approach proposes an important intervention to reduce the risk of AC among high-risk populations. (5) Although AC screening is recommended for PLWH; research suggests that lack of knowledge and awareness about HPV infection and AC risk-factors could influence the uptake of AC screening.(8–10) Therefore, this study aimed to assess awareness and knowledge of HPV infection, AC risk-factors, and perceived risk among PLWH in PR.

## METHODS

### Study design and population.

Data from a cross-sectional study conducted from November 2020 to December 2021 was analyzed. Participants had to be: 1) PLWH, 2) aged 26 years and older, and 3) living in PR. Potential participants were identified through social media, on-site clinic promotion with research assistants, flyers/banners at immunologic clinics, TV and radio advertising, and participants’ word of mouth. Of the 370 possible candidates contacted, 290 were interested in participating, 268 were eligible, and 212 (79%) PLWH participated in the study. Individuals who consented completed a telephone interview where information about sociodemographic, lifestyle, and clinical characteristics was gathered. This study was approved by the Institutional Review Board of the University of Puerto Rico Comprehensive Cancer Center (2019-07-10).

### Data collection.

**HPV awareness** was dichotomized (Yes/No). The “Yes” category included participants who answered “yes” to the question “*Before today, have you ever heard of HPV infection?”;* the “No” categoryincluded those who answered “no” or “do not know”.

To evaluate **HPV and AC knowledge**, two 13-item scales were constructed. The **HPV infection knowledge** scale included questions regarding risk-factors for HPV infection, and HPV-associated cancers, among others. The **AC risk-factors knowledge** scale included questions regarding HIV, tobacco use, HPV infection, and anal sex (See table 3).(11) All questions could be answered as: “Yes”, “No”, “Do not know”, and “Refuse to answer”. These answers were then coded as correct or incorrect (Don’t know answers were classified as incorrect). For both scales, responses were summed and divided by the number of questions (n=13). Participants who scored ≥70% were classified as having adequate knowledge whereas those who scored <70% were classified as not having adequate knowledge.

### Statistical analysis

Frequencies were used to describe categorical variables, whereas central tendency measures were used for continuous variables. Chi-square statistics and Fisher exact test were used to assess the association between HPV infection knowledge and AC risk-factors and the other covariates. Logistic regression models using generalized linear models were used to determine the association between HPV infection awareness, HPV infection knowledge, and AC risk-factors knowledge, and the potential predictors that were statistically significant in the bivariate analysis (p<0.05). The Likelihood Ratio test assessed the interaction terms between the predictor variables. If an interaction term had a p-value of less than 0.05, the model was stratified by this variable. When no interaction terms were seen, the models were adjusted according to sexual risk group and age. Stata for Windows, version 17 (Stata Corporation, College Station, TX) was used for the statistical analysis.

## RESULTS

The median age of the participants was 54 years (IQR: 46, 58). The majority (67.5%) reported being assigned male sex at birth, having an income less than $20,000 (71.7%), an education level of high school or more (54.3%), and having government health insurance (84.4%). Furthermore, 44% of the participants were MSM, 33% were women and 23% were men who have sex with women (MSW). The median years for living with HIV was 17 years (IQR: 9–25). Almost all participants reported using antiretroviral therapy (99.1%), and 20.8% reported having a history of acquired immunodeficiency syndrome (AIDS).

The self-reported prevalence of HPV infection was 12.0%. Although awareness of HPV vaccination was 53.8%, only 7.6% of the participants reported being vaccinated against HPV infection. More than half of the participants reported a history of sexually transmitted infections (STIs) (63%), while none reported having a history of AC. Regarding lifestyle characteristics, 29.3% reported being current smokers, 55.2% reported current alcohol intake and 64.1% reported use of non-IV drugs in their lifetime. Furthermore, more than half (80.2%) perceived having excellent/very good or good health. (Table 1)

HPV awareness was high among the study population (82.1%), but only 40.2% had adequate HPV knowledge (score ≥70). The majority correctly answered items about the risk of acquiring HPV; HPV can be transmitted by sexual contact (89.1%), having sex with multiple partners (95.4%), partners with multiple sexual partners (91.4%), or having sex at early ages (71.8%). When asked about HPV-related cancers, most of the participants correctly identified cervical cancer (87.4%), AC (83.9%) and oral cancer (67.2%). More than half think that HPV is uncommon (66.1%), that HPV-infected people always present symptoms (66.1%), and that genital warts are caused by HPV (62.6%). On the contrary, less than half think that condoms always prevent HPV infection (43.7%), and that HPV can disappear without treatment (8.6%).

Regarding AC, only 3.8% of the study population had adequate knowledge of AC risk-factors. However, a high proportion of participants correctly identified number of sex partners (87.3%), HPV infection (81.6%), unprotected sex (78.3%), anal sex (76.4%), HIV (69.8%), smoking (66.5%), and having gynecologic cancers (62.4%) as risk-factors for AC. Most felt they were at higher risk of AC than other people of the same age (51.4%). Some of the participants thought that screenings for AC are uncomfortable (40.0%), most of them had anal Pap (52.2%) history and the majority (59.6%) had done the anal Pap in the last 12 months. Additionally, 76.9% have considered getting tested for AC, and 96.7% indicated they will do the anal Pap test in the future (Data not shown). However, a history of high-resolution anoscopy (HRA) uptake was less common (19%) in this population.

### Bivariate analysis

**HPV awareness** was significantly higher among women (89.9%), people with a history of anal Pap uptake (89.0%), and men who have sex with men (83.9%) (p<0.05). No other significant differences were seen. (Table 2)

Higher **HPV knowledge** adequacy was observed in participants with an education level of more than high school (51.6% vs 26.0%) and with a history of HPV infection (65.2% vs 35.8%). Also, participants with a history of sexually transmitted infections (47.8% vs 26.2%) and with a self-reported health perception of excellent/very good or good (45.6% vs 21.1%) showed more knowledge about HPV infection (p<0.05). (Table 4)

An association was seen between having adequate knowledge of **AC risk-factors** and the years living with HIV. More specifically, 6.8% of individuals living with HIV for 15 years or more had adequate knowledge of AC as compared to 0.0% of those who had lived with HIV for less time (p<0.05). (Table 4)

### Multivariate analysis

Given significant interactions with age, logistic regression models were stratified by age-group (Table 5). Among PLWH aged 50 years or older, MSM (OR: 1.26, 95% CI: 1.07–1.47) and women (OR: 1.35, 95% CI: 1.15–1.59) had higher odds of **HPV awareness** than MSW in that age group. Among PLWH under 50 years, those with a history of anal Pap test had higher odds of HPV awareness (OR 1.34, 95% CI: 1.08–1.66) compared to their counterparts.

In multivariate logistic regression models, participants with an education level of more than high school (OR: 1.28, 95% CI: 1.10–1.50) and with a history of HPV diagnosis (OR: 1.33, 95%CI: 1.08–1.65) were more likely to have adequate **HPV knowledge** compared to their counterparts. In addition, PLWH who reported having good/very good/excellent health were more likely to have adequate knowledge as compared to those who reported poor/regular health (OR: 1.23, 95% CI: 1.03–1.47). Although not statistically significant, participants with a history of STIs (OR: 1.13, 95% CI: 0.97–1.32) were also more likely to have adequate HPV knowledge. (Table 6)

For **knowledge of AC risk-factors**, PLWH for 15 years or more had increased odds of having adequate knowledge (OR: 1.08, 95% CI: 1.02–1.14) compared to those diagnosed more recently (data not shown).

## DISCUSSION

Awareness and education play a pivotal role in adopting healthy behaviors and facilitating opportune detection and management of acute conditions like AC.(12, 13) Therefore, cancer control and prompt detection are directly related to risk-factors awareness, knowledge, and improved likelihood of survival(14, 15). Thus, populations at increased risk of developing cancer must be aware of risk-factors to adopt protective behaviors. It is known that PLWH has a major risk to have HPV infection and in consequence AC, hence awareness and education about HPV and AC risk-factors are imperative among this population.

The principal aim of this study was to assess awareness and knowledge of HPV infection and AC risk-factors among PLWH in PR. The results showed that although PLWH were aware of HPV, their knowledge about this condition was limited. Likewise, results are consistent with studies completed by Feeney, et al. (8) and Adegboyega et al.(16), where poor knowledge and awareness of HPV were described in HIV-positive and negative gay and bisexual men and African Americans and African immigrants’ young adults, respectively. Nevertheless, according to results from our study in PLWH living in PR, education, previous history of HPV infection and STIs seem to positively influence HPV infection and AC knowledge, similar to other studies where a higher education(17–19) is related to higher knowledge about HPV infection and AC. Similarly, another study among high-risk men of Hispanic living in PR showed that a history of STIs was associated with a higher knowledge of HPV infection. (20) Thus, previous experiences should be considered as they can influence knowledge and screening aptitudes.(21) Another important finding of our study was that knowledge of HPV infection was low among participants who perceived having poor or regular health as compared to the study of Gillis et.al.(22) where self-perceived risk influenced HPV-related knowledge. This suggests that the perception of poor health could become a barrier to the interest in obtaining more knowledge about HPV.

Equally important, although 40.3% had adequate knowledge of HPV infection and 53.8% of participants were aware of HPV vaccination, only 7.5% of participants were vaccinated. Contrary, in the study of Amboree et al. they reported that PLWH had lower knowledge of HPV besides a higher vaccine uptake. (23) They assume that this could be related to their engagement to care. Meanwhile, in studies of women(24) and men(25) living with HIV in Canada, a lower HPV vaccine uptake was reported while they demonstrated engagement with regular care. (25)

On the other hand, limited knowledge about AC risk-factors was reported by the participants of this study. This is similar to the studies of Rodriguez et al. and Wells et al., that show a lack of awareness and knowledge about AC risk in women living with HIV and HIV-negative women living in the US.(9, 18) Also, other studies among gay and bisexual men and young men living in the US reported a lack of information about AC.(19, 23) Contrariwise, participants with more than 15 years of having the diagnosis of HIV, demonstrate more knowledge about AC risk-factors presenting the possibility that PLWH are more expose to health education and medical resources, leading to more awareness or their experiences stimulated them to become informed about potential complications like AC.

Study findings suggest a challenge for primary care professionals because health illiteracy has been identified as a main barrier to cancer prevention, affecting the engagement of PLWH with preventive activities.(26) Innovative preventive methods are needed for early detection and the improvement of AC management.(27) These efforts should also include the importance of HPV vaccination, since although the majority of subjects were aware of HPV vaccination, less than 10% were vaccinated. Primary care professionals are considered a thrust source of information (26) thus there is a big responsibility to train this population with the latest information about HPV infection, AC risk-factors and prevention strategies.

## STUDY STRENGTH AND LIMITATIONS

Our study is one of the few to evaluate HPV awareness and knowledge and AC risk-factors knowledge among a Hispanics living with HIV. Results underscore the need for increased awareness and knowledge about HPV and AC among high-risk populations. Limitations include self-reported nature of the data, which could lead to information bias, and the use of a convenience sample, limiting the generalizability of the findings to all PLWH in Puerto Rico.

## CONCLUSIONS

Despite high awareness of HPV, limited knowledge about HPV and AC risk factors were observed among this Hispanic population of PLWH living in PR, underscoring the need for educational efforts within this population as an AC prevention strategy. Although PLWH are at a higher risk of developing AC, our study revealed that few individuals have adequate knowledge of AC; despite that more than half of the participants were up to date with AC screening recommendations (having undergone anal Pap test during the last 12 months). Additionally, despite awareness of the HPV vaccine, less than 10% were vaccinated against it. Educational efforts should focus on the importance of AC screening and HPV vaccination, as well as on the risk-factors for cancer, particularly among PLWH.

## Figures and Tables

**Table 1.** Characteristics of the Study Population (n=212).

Variables	n	%
**Sociodemographic**		
**Sex at birth**		
Men	143	67.5
Women	69	32.6
**Age (years)**		
< 50	69	32.6
≥ 50	143	67.5
**Education**		
High school or less	97	45.8
More than high school	115	54.3
**Civil Status**		
Single	104	49.1
Married/consensual	62	29.3
Separate/Widowed	46	21.7
**Annual income, US $**		
< 20,000	152	71.7
≥ 20,000	60	28.3
**Health insurance** ^a^		
None	5	2.4
Public	178	84.4
Private	28	13.3
**Sexual Risk group** ^a^		
MSM	93	44.1
MSW	49	23.2
Women	69	32.7
**Lifestyle**		
**Current Smoking**		
No	150	70.8
Yes	62	29.3
**Current Alcohol Uptake**		
No	95	44.8
Yes	117	55.2
**Non-injectable drug use (Lifetime)**		
No	76	35.9
Yes	136	64.1
**Injectable drug use (Lifetime)**		
No	191	90.5
Yes	20	9.5
**Clinical**		
**Years living with HIV**		
< 15	94	44.3
≥ 15	118	55.7
**Antiretroviral intake**		
No	2	0.9
Yes	210	99.1
**AIDS history**		
No	168	79.3
Yes	44	20.8
**History of previous anal Pap** ^b^		
No	100	47.9
Yes	109	52.2
**Last anal Pap (n=109)**		
1–12 months	65	59.6
13 months	44	40.4
**History of previous HRA** ^c^		
No	166	81.0
Yes	39	19.0
**HPV vaccination awareness**		
No	98	46.2
Yes	114	53.8
**HPV vaccination uptake**		
No	196	92.4
Yes	16	7.6
**History of HPV (Self-reported)** ^b^		
No	184	88.0
Yes	25	12.0
**History of STIs**		
No	78	36.8
Yes	134	63.2
**Perceived Health**		
Poor/Regular	42	19.8
Excellent/Very good/Good	170	80.2
**HPV awareness**		
No	38	17.9
Yes	174	82.1
**HPV knowledge (n=174)** ^d^		
<70	104	59.8
≥70	70	40.2
**AC risk-factors knowledge**		
<70	204	96.2
≥70	8	3.8

Missing values:

a 1;

b 3;

c 7;

d HPV knowledge was evaluated only for those participants who mentioned having heard about HPV

**Table 2.** Factors associated with HPV awareness among PLWH (n=212).

	HPV awareness		
Variables	No n(%)	Yes n(%)	P
**Sociodemographic**			
**Sex at birth**			
Female	7 (10.1)	62 (89.9)	**0.040**
Male	31 (21.7)	112 (78.3)	
**Age (years)**			
<50	14 (20.3)	55 (79.7)	0.533
>50	24 (16.8)	119 (83.2)	
**Education**			
High school or less	20 (20.6)	77 (79.4)	0.348
More than high school	18 (15.7)	97 (84.4)	
**Annual Income US $**			
>20,000	29 (19.1)	123 (81.0)	0.485
<20,000	9 (15.0)	51 (85.0)	
**Health insurance** ^a^			
None	0 (0.0)	5 (100.0)	0.621
Public	32 (18.0)	146 (82.0)	
Private	6 (21.4)	22 (78.6)	
**Sexual risk group** ^a^			
MSW	16 (32.7)	33 (67.4)	
MSM	15 (16.1)	78 (83.9)	**0.006**
Women	7 (10.1)	62 (89.9)	
**Lifestyle**			
**Current Smoking**			
No	26 (17.3)	124 (82.7)	0.727
Yes	12 (19.4)	50 (80.7)	
**Current Alcohol Uptake**			
No	18 (19.0)	77 (81.0)	0.726
Yes	20 (17.1)	97 (82.9)	
**Non-injectable drug use (Lifetime)**			
No	11 (14.5)	65 (85.5)	0.327
Yes	27 (19.8)	109 (80.2)	
**Injectable drug use (Lifetime)**			
No	33 (17.3)	158 (82.7)	0.392
Yes	5 (25.0)	15 (75.0)	
**Clinical**			
**Years living with HIV**			
<15 years	21 (22.3)	73 (77.7)	0.135
>15 years	17 (14.4)	101 (85.6)	
**AIDS history**			
No	29 (17.3)	139 (82.7)	0.623
Yes	9 (20.5)	35 (79.6)	
**History of HPV diagnosis**^**d**^ **(Self-reported)**			
No	36 (19.6)	48 (80.4)	0.160
Yes	2 (8.0)	23 (92.0)	
**History of previous anal Pap** ^b^			
No	25 (25.0)	75 (75.0)	**0.008**
Yes	12 (11.0)	97 (89.0)	
**Last Pap (n=109)**			
1–12 months	9 (13.9)	56 (86.2)	0.250
13 months	3 (6.8)	41 (93.2)	
**History of previous HRA** ^c^			
No	31 (18.7)	135 (81.3)	0.631
Yes	6 (15.4)	33 (84.6)	
**History of STIs**			
No	17 (21.8)	61 (78.2)	0.262
Yes	21 (15.7)	113 (84.3)	
**Health perception**			
Poor/Regular	4(9.5)	38 (90.5)	0.113
Excellent/Very good/Good	34 (20.0)	136 (80.0)	

Missing values:

a 1;

b 3;

c 7

**Table 3.** Knowledge about HPV and AC among PLWH in Puerto Rico.

HPV knowledge items (n=174)		
Correct items^a^	n	(%)
Sex with multiple partners increases the risk to contract HPV	165	95.4
Partners with multiple sexual partners increase the risk to contract HPV	159	91.4
HPV can be transmitted by sexual contact	155	89.1
HPV can cause cervical cancer	152	87.4
HPV can cause AC	146	83.9
Sex at an early age increases the risk to contract HPV	125	71.8
HPV can cause oral cancer	117	67.2
Genital warts are caused by HPV	109	62.6
HPV disappears without treatment	15	8.6
**Incorrect items** ^b^		
HPV infected people always know that they are infected	147	85.0
HPV is not a common infection	115	66.1
HPV infected people always present symptoms	115	66.1
Condoms always prevent HPV infection	76	43.7
**AC risk-factors items (n=212)**		
**Correct items** ^a^		
Multiple sexual partners is a risk-factor for AC	185	87.3
HPV is a risk-factor for AC	173	81.6
Not using condoms is a risk-factor for AC	166	78.3
Anal sex is a risk-factor for AC	162	76.4
HIV is a risk-factor for AC	148	69.8
Smoking is a risk-factor for AC	141	66.5
History of gynecologic cancers is a risk-factor	131	62.4
Having sex at early age is a risk-factor	88	41.5
**Incorrect items** ^b^		
AC is more common in men than women	67	31.6
Alcohol uptake is a risk-factor	51	24.1
Bad family genetic profile is a risk-factor for anal AC	38	17.9
Bad diet is a risk-factor for AC	36	17.0
Dangerous chemicals in food and water are a risk-factor for AC	30	14.2

a These are true statements about HPV and AC risk knowledge and proportion of participants that identified these statements correctly.

b These are false statements about HPV bout HPV and AC risk knowledge and proportion of participants that identified these statements correctly.

**Table 4.** Factors associated to knowledge of HPV infection and AC risk-factors among PLWH in Puerto Rico.

Variables	HPV infection knowledge (amongthose aware of HPV) (n=174)			AC risk-factors knowledge (n=212)		
	Score <70 n (%)	Score ≥70 n (%)	p-value	Score <70 n (%)	Score ≥70 n (%)	p-value^Δ^
**Sociodemographic**						
**Sex at birth**						
Female	41(66.1)	21(33.9)	0.203	66(95.7)	3(4.4)	0.717
Male	63(56.2)	49(43.8)		138(96.5)	5(3.5)	
**Age (years)**						
<50	32(58.2)	23(41.8)	0.771	67(97.1)	2(2.9)	1.000
≥50	72(60.5)	47(39.5)		137(95.8)	6(4.2)	
**Education**						
High school or less	57(74.0)	20(26.0)	**0.001**	94(96.9)	3(3.1)	0.729
More than high school	47(48.5)	50(51.6)		110(95.7)	5(4.4)	
**Annual Income US $**						
<20,000	73(59.4)	50(40.7)	0.861	146(96.1)	6(4.0)	1.000
≥20,000	31(60.8)	20(39.2)		58(96.7)	2(3.3)	
**Health Insurance** ^a^						
Public	88(60.3)	58(39.7)	1.000	170(95.5)	8(4.5)	0.672
Private	13(59.1)	9(40.9)		28(100.0)	0(0.0)	
None	3(60.0)	2(40.0)		5(100.0)	0(0.0)	
**Sexual risk group** ^a^						
MSM	40(51.3)	38(48.7)	0.094	89(95.7)	4(4.3)	0.812
MSW	23(69.7)	10(30.3)		48(98.0)	1(2.0)	
Women	41(66.1)	21(33.9)		66(95.7)	3(4.4)	
**Lifestyle**						
**Current smoking**						
No	73(58.9)	51(41.1)	0.703	144(96.0)	6(4.0)	1.000
Yes	31(62.0)	19(38.0)		60(96.8)	2(3.2)	
**Current alcohol intake**						
No	46 (59.7)	31 (40.3)	0.994	91 (95.8)	4(4.2)	1.00
Yes	58 (59.8)	39 (40.2)		113 (96.6)	2(3.4)	
**Non-injectable drug use (Lifetime)**						
No	42 (64.6)	23 (35.4)	0.314	73 (96.0)	3 (4.0)	1.00
Yes	62 (56.9)	47 (42.1)		131 (96.3)	5 (3.7)	
**Injectable drug use (Lifetime)**						
No	92 (58.2)	66 (41.8)	0.255	184 (96.3)	7 (3.41)	0.556
Yes	11 (73.3)	4 (26.7)		19 (95.0)	1 (5.0)	
**Clinical**						
**Years with HIV**						
<15	44(60.3)	29(39.7)	0.908	94(100.0)	0(0.0)	**0.010**
≥15	60(59.4)	41(40.6)		110(93.2)	8(6.8)	
**AIDS diagnosis**						
No	85(61.2)	54(38.9)	0.459	162(96.4)	6(3.6)	0.672
Yes	19(54.3)	16(45.7)		42(95.5)	2(4.6)	
**History of HPV (Self-reported)** ^b^						
No	95(64.2)	53(35.8)	**0.007**	178(96.7)	6(3.3)	0.596
Yes	8(34.8)	15(65.2)		24(96.0)	1(94.0)	
**History of previous anal Pap** ^c,d^						
No	50(66.7)	25(33.3)	0.144	96(96.0)	4(4.0)	1.000
Yes	54(55.7)	43(44.3)		105(96.3)	4(3.7)	
**Last Anal Pap (n=109)**						
1–12 months	30(53.6)	26(46.4)	0.627	62(95.4)	3(4.6)	0.646
13 months	24(58.5)	17(41.5)		43(97.7)	1(2.3)	
**History of previous HRA** ^e,f^						
No	82(60.7)	53(39.3)	0.516	160(96.4)	6(3.6)	1.000
Yes	18(54.55)	15 (45.5)		38(97.4)	1(2.6)	
**STIs infection history**						
No	45(73.8)	16(26.2)	**0.006**	74 (94.9)	4 (5.1)	0.470
Yes	59(52.2)	54(47.8)		130 (97.0)	4 (3.0)	
**Health perception**						
Poor/Regular	30 (79.0)	8 (21.1)	**0.006**	42 (100.0)	0 (0.0)	0.361
Excellent/Very good/Good	74 (54.4)	62 (45.6)		162 (95.3)	8 (4.7)	

Δ Fisher exact test; Missing values:

a 1;

b 3

c 2;

d 3;

d 2,

e 6;

f 7

**Table 5.** Logistic regression models of factors associated to HPV infection awareness stratified by age.

	HPV infection awareness Crude OR (95% CI)		HPV infection awareness Adjusted OR (95% CI)	
**Age-Groups (years)**	<50	≥50	<50	≥50
**History of previous anal Pap**				
No	1.0	1.0	1.0	1.0
Yes	**1.26 (1.04–1.52)**	1.11 (0.99–1.26)	**1.34 (1.08–1.66)**	1.03 (0.91–1.17)
**Sexual risk group**				
MSW	1.0	1.0	1.0	1.0
MSM	0.95 (0.73–1.23)	**1.30 (1.12–1.50)**	0.81 (0.62–1.06)	**1.26 (1.07–1.47)**
Women	0.91 (0.73–1.23)	**1.39 (1.20–1.62)**	0.82 (0.61–1.11)	**1.35 (1.15–1.59)**

Sample size of crude OR- History of previous anal Pap: <50 (n=69); ≥50 (n=140); Sexual risk group - <50 (n=68); ≥50 (n=143).

**Table 6.** Logistic regression models of factors associated to adequate HPV knowledge.

	Adequate HPV knowledge Crude OR (95% CI) (n=174)	Adequate HPV knowledge Adjusted OR (95% CI) (n = 170)
**Age (years)**		
< 50	1.0	1.0
≥ 50	0.98 (0.83–1.14)	1.03 (0.89–1.19)
**Sexual risk group** ^a^		
MSW	1.0	1.0
MSM	1.20 (0.99–1.47)	1.01 (0.83–1.24)
Women	1.04 (0.84–1.27)	1.04 (0.85–1.27)
**Education**		
High school or less	1.0	1.0
More than high school	**1.29 (1.12– 1.49)**	**1.28 (1.10–1.50)**
**History of HPV infection** ^b^		
No	1.0	1.0
Yes	**1.34 (1.09–1.66)**	**1.33 (1.08–1.65)**
**History of STIs**		
No	1.0	1.0
Yes	**3.36 (1.34–8.45)**	1.13 (0.97–1.32)
**Health Perception**		
Poor/Regular	1.0	1.0
Good/Very good/Excellent	**1.28 (1.07–1.52)**	**1.23 (1.03–1.47)**

Missing values:

a 1;

b 3

## Data Availability

The data used during the current study is available from the corresponding author on reasonable request. Data is not publicly available due to containing information that could potentially compromise the privacy of the participants.

## Abbreviations

PLWH: People living with human immunodeficiency virus
HPV: Human papillomavirus
PR: Puerto Rico
US: United States
AC: Anal cancer
Pap: Papanicolaou
MSM: Men who have sex with men
IANS: International Anal Neoplasia Society
MSW: Men who have sex with women
AIDS: Acquired immunodeficiency syndrome
STIs: Sexually transmitted infections
HRA: High-resolution anoscopy
